# A specific tongue microbiota signature is found in patients displaying an improvement of orosensory lipid perception after a sleeve gastrectomy

**DOI:** 10.3389/fnut.2022.1046454

**Published:** 2023-01-11

**Authors:** Arnaud Bernard, Loredana Radoi, Jeffrey Christensen, Florence Servant, Vincent Blasco-Blaque, Séverine Ledoux, Xavier Collet, Philippe Besnard

**Affiliations:** ^1^UMR 1231 INSERM/Univ Bourgogne Franche-Comté/AgroSup Dijon, Dijon, France; ^2^Médecine Bucco-dentaire, Hôpital Louis Mourier (APHP-Nord), Colombes, France & Univ Paris Cité/CESP-UMR 1018 INSERM, Paris, France; ^3^UMR 1297 INSERM/Univ Toulouse III Paul Sabatier, Toulouse, France; ^4^Vaiomer, Toulouse, France; ^5^Explorations Fonctionnelles, Hôpital Louis Mourier (APHP-Nord), Colombes, France

**Keywords:** fat taste, bariatric surgery, oral microbiota, circumvallate papillae, eating behavior

## Abstract

**Introduction:**

A preferential consumption of low-fat foods is reported by most of the patients after a vertical sleeve gastrectomy (VSG). The fact that a recent study shed light on a relationship between oral microbiota and fat taste sensitivity in obese patients prompted us to explore whether such a connection also exists in the context of a VSG.

**Methods:**

Thirty-two adult female patients with a severe obesity (BMI = 43.1 ± 0.7 kg/m^2^) and candidates for a VSG were selected. Oral microbiota composition surrounding the gustatory circumvallate papillae (CVP) and the lipid perception thresholds were explored before and 6 months after surgery.

**Results:**

VSG was found to be associated both with a qualitative (compositional changes) and quantitative (lower gene richness) remodeling of the peri-CVP microbiota. Analysis of the lipid perception allowed us to distinguish two subgroups: patients with a post-operative improvement of the fat taste sensitivity (*i.e.*, with a lower threshold, *n* = 14) and unimproved patients (*n* = 18). Specific peri-CVP microbiota signatures also discriminated these two subgroups, unimproved patient being characterized by higher levels of *Porphyromonas, Fusobacterium*, and *Haemophilus* genera associated with lower levels of *Atopobium* and *Prevotella* genera as compared to the lipid-improved patients.

**Conclusion:**

Collectively, these data raise the possibility that the microbial environment surrounding gustatory papillae might play a role in the positive changes of fat taste sensitivity observed in some patients after VSG.

## Introduction

Obesity is associated with multiple metabolic disorders at the origin of serious co-morbidities including type-2 diabetes, hypertension, or cancer. Recent changes in our food supply with, among other things, an easy access to low-cost tasty energy-dense foods rich in fat and sugar is thought to be one of major environmental contributors to the dramatic rise of obesity prevalence worldwide. A preferential consumption of high palatable foods (*i.e.*, pleasant in mouth), rich in fat is also frequently observed in obese subjects (1–3). This eating behavior suggests that obesity *per se* might also induce food mouthfeel changes able to modify the food choice habits. An implication of the gustatory pathway in this eating pattern change is likely. Indeed, obesity alters gene expression in human taste buds (4) and reduces the abundance of gustatory fungiform papillae in mice and humans (5). Moreover, functional alterations of brain areas involved both in the taste perception and the reward processing have been identified in subjects with obesity [for review, see (6, 7)]. Consistent with this assumption, a reduction of the orosensory perception of fat stimuli associated with an overconsumption of lipid-rich foods was found in some patients with obesity (8).

Obesity surgery is an efficient method to induce a long-term body weight loss and to correct obesity-associated metabolic dysfunctions in subjects with a morbid obesity (BMI ≥ 40 kg/m^2^) (9). Among the different bariatric procedures developed, the vertical sleeve gastrectomy (VSG) consists to remove two/third of the stomach. By combining efficient weight and metabolic benefits with a low post-operative complication rate, VSG is actually the most popular bariatric surgery worldwide (10). Interestingly, most of the patients having undergone a VSG report a change of their flavor perception associated with a reduced interest for high-fat foods (11). This healthier eating behavior suggests that VSG could reverse the obesity-mediated decrease of the orosensory perception of lipids, at least of some subjects. Since no systematic relationship between the BMI loss and the improvement of fat taste sensitivity is observed in humans (12), the underlying mechanism remains elusive.

The oral cavity is one of the most abundant and diversified microbial community within the body (13). The greatest bacteria abundance is found upon the dorsal tongue where microbial communities harbor a very complex spatial pattern due to specific ecological niches (14). Interestingly, the circumvallate papillae (CVP), housing most of the oral taste buds, exhibit a dome-shape structure with a circular cleft which could favor the development of specific bacterial communities with the potential to affect the host cell functions. Consistent with this assumption, an impairment to sense lipids was found in obese subject displaying a specific CVP microbiota (15).

Collectively, these observations raise a basic question: is there a relationship between the peri-papillae microbiome and the orosensory lipid perception after VSG? To explore this issue, oral microbiota samples in the direct vicinity of CVP were analyzed and the lipid perception thresholds were explored in volunteers with a severe obesity before and 6 months after surgery. Two complementary end-points have been investigated: the impact of VSG on the CVP microbiota composition and the relationship between CVP microbiota and fat taste sensitivity using the 3-alternative force-choice procedure (3-AFC) (16).

## Subjects and methods

### Subjects

The present clinical study is a part of the HumanFATaste2 program approved by French Ethic Committees (Comité de Protection des Personnes, CPP n° 15-032 and Agence Nationale de la Sécurité des Médicaments, ANSM n° 150811B-21) and registered at Clinical Trials (NCT#02497274). Forty-four patients with severe obesity (BMI ≥ 40 kg/m^2^) were included in the HumanFATaste2 program (17). Among them, 32 patients have undergone a VSG and were included in the present work. Inclusion criteria were the following: women, aged 18 to 55 years, candidate for bariatric surgery based on criteria established by international experts (BMI > 40 or >35 kg/m^2^ with severe comorbidities, after failure of multidisciplinary care for a period of at least 1 year); and affiliation to a health insurance. The exclusion criteria were the following: taking medications known to alter taste, tooth decays, previous surgical treatment of obesity, diabetes (hypoglycemic drug intake or fasted plasma glucose level > 7 mmol/L), malignant or chronic inflammatory diseases and tobacco use, alcohol or drugs abuse. All subjects received detailed information about the study and provided a written consent. To avoid gender-mediated bias (18) and inter-individual variability, this study was conducted on women only, each patient being her own control (before and after VSG).

**FIGURE 1:**
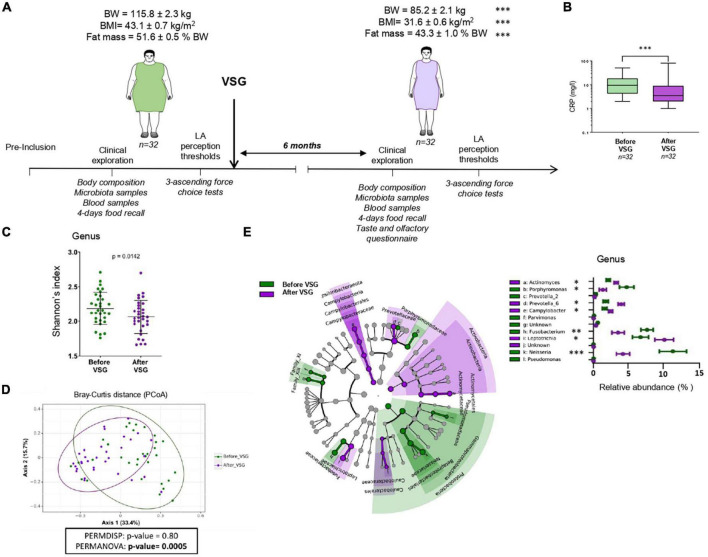
Vertical sleeve gastrectomy (VSG)-associated changes in the composition of body and tongue coating microbiota surrounding the circumvallate gustatory papillae (CVP). **(A)** Time course of experiment and related analyses. **(B)** Plasma C-reactive protein (CRP) levels before and after VSG (Wilcoxon paired test). **(C)** Alpha-diversity of microbiota surrounding CVP (Shannon index at the genus level). **(D)** Beta-diversity of microbiota surrounding CVP before vs. after VSG using principal coordinates analysis plot (PCoA) on Bray-Curtis distance and PERMANOVA test. Ellipses indicate 95% confidence intervals of multivariate *t*-distribution around centroids of groups. **(E)** Taxonomic LEfSe analysis of VSG impacts (before VSG vs. after VSG) on CVP microbiota. Cladogram of taxonomic assignments from 16S rRNA gene sequence data. Taxa are shaded according to the group in which it is most abundant. Histogram showing the percent relative abundance with corresponding letters in the cladogram. BW, body weight; LA, linoleic acid. Means ± SEM. *p < 0.05; ^**^p < 0.01; ^***^p < 0.001.

Vertical sleeve gastrectomy was performed laparoscopically, as previously described (19). Pre- and post-operative multidisciplinary managements were performed at the Louis Mourier Hospital (Assistance publique hôpitaux de Paris–APHP) according to French recommendations of the Haute Autorité de Santé (HAS) (20). Analysis of the body composition including fat mass using multifrequence impedancemetry (Seca, France), peri-papillae microbiota samples, blood collection and questionnaire on dietary habits (4-days food recall) were performed before and 6 months after VSG (Figure 1A). Plasma C-reactive protein (CRP) levels, used as early biomarker of inflammation, were assessed by using a ELISA kit (Enzo Life sciences, France) in the same laboratory for all subjects. Six months after VSG, patients have to assess their change in gustatory and olfactory perception [taste and olfaction changes (TOC) questionnaire (21)], adapted to include questions about fat-mediated sensations. An analogic scale scored from 1 (no change) to 10 (maximal change) was used to evaluate the subjective gustatory and olfactory perception.

### Oral microbiota samples and analysis

To delineate whether VSG is associated with changes in the gustatory papillae microbiota ecology, swab samples before and 6 months after surgery were taken from each participant. Oral microbiota samples were taken in the morning in overnight fasted subjects directly from the V-shaped row of the CVP located at the back of the tongue dorsa by smear (20 s) with a sterile swab according to the previously published procedure (15). Subjects were instructed not to brush their teeth or use mouthwash in morning prior to oral swab in order not to modify the oral flora. The bacterial composition before and after surgery was determined by Vaiomer (Toulouse-Labège, France) by sequencing variable regions of the 16S rRNA bacterial gene. The V3-V4 hyper-variable regions of the 16S rRNA gene were amplified from the DNA extracts during a first PCR step using universal 16S primers V2 (Vaiomer, Toulouse-Labège, France). The expected amplicon lengths were between 350 and 500 base pairs (bp). For each sample, a sequencing library was generated by addition of sequencing adapters and multiplexing indexes during a second PCR step before sequencing on an Illumina MiSeq machine in *2* × *300 bp* paired-end reads mode as described previously (22, 23).

The targeted metagenomic sequences from microbiota were analyzed using the bioinformatics pipeline established by Vaiomer to find operational taxonomic units (OTUs) with Galaxy solution (FROGS) guidelines (24). Briefly, after demultiplexing of barcoded Illumina paired reads, single-read sequences were cleaned, the last 10 and 20 bases of respectively, R1 and R2 reads were trimmed and paired into longer fragments. OTUs were produced with single-linkage clustering using two passes of the FROGS embedded swarm algorithm: the first pass with an aggregation distance equal to 1 and the second pass with an aggregation distance equal to 3. The taxonomic assignment was performed by BLAST against SILVA 132 database to determine bacterial profiles from phylum to genus, and when reachable to species level. The following filters were applied: amplicons with a length below 350 nt or a length above 500 nt were removed and OTUs with abundance lower than 0.005% were removed. Alpha and beta diversity analyses were conducted on the OTU table.

The functional metagenome was inferred from the clustered 16S sequences using the PICRUSt software (version 1.0.0) as per the instructions provided for the Genome Prediction Tutorial for PICRUSt^1^ with recommended scripts and default settings (25). As described in the PICRUSt tutorial, the sequences previously grouped into OTUs were processed through the QIIME closed reference OTUs picking tool with a 97% similarity threshold to obtain a set of OTUs IDs from the Greengenes reference collection (gg_otus_13_5.tar.gz) as input for prediction of corresponding metagenomes by PICRUSt. Through this inference process, the abundance values of each OTU were normalized to their respective predicted 16S rRNA gene copy numbers and then multiplied by the respective gene counts for metagenome prediction. PICRUSt was also used to calculate the nearest sequenced taxon index (NSTI) to quantify dissimilarity between reference genomes and the predicted metagenomes. The resulting core output was a list of Kyoto encyclopedia of genes and genomes (KEGG) orthologues and predicted gene count data for each sample. We used in house scripts to parse the output into KEGG module categories for functional pathways and structural complex hierarchies using the KEGG database.^2^ The output matrix containing the relative abundance of KEGG orthologous groups (KO) per sample was processed with the online Galaxy interface for linear discriminant analysis effect size (LEfSe) using an alpha parameter significance threshold for the Kruskal–Wallis test among classes set to 0.05 and the logarithmic linear discriminant analysis (LDA) score cut-off was set to 2.0. Respective cladograms were generated with modules at the lowest level. Quantitative plots of differential features were generated from normalized module level predicted gene data showing means with standard deviation using GraphPad Prism 6 software (GraphPad Software, La Jolla, CA, USA).

### Oral LA-threshold perception

The orosensory perception of lipids was determined at 9 a.m. in fasting patients using the 3-AFC method with the linoleic acid (LA) as lipid model (15). LA was chosen because this polyunsaturated long-chain fatty acid, widely found in foods, binds with a high affinity the lipid receptors expressed in the taste buds (*i.e.*, CD36 and GPR120) (26). Patients checked (sip and spit) sets of three samples (two with the control solution, one with LA in the control solution), and had to identify the sample that was different from the two others. Sets were presented in an ascending concentration (from 0.00028 to 5% LA) spaced by 0.25 log units (18 solutions in total). The procedure was stopped when the LA sample was correctly identified three times consecutively. This LA concentration represented the perception threshold value of the patient.

The protocol used to prepare LA solution is fully detailed elsewhere (8, 16). In brief, LA (Sigma Aldrich, France) oil-in water emulsions were prepared in a solution of 5% acacia gum (wt/wt; Fluka, USA), 5% mineral oil (wt/wt; Cooper, France), and 0.01% EDTA (wt/wt; VWR international, USA) diluted in mineral water. Acacia gum and paraffin oil were added to limit viscosity and lubricity differences between control and experimental samples. EDTA was added to prevent the oxidation of LA (16). Samples were mixed conventionally by using a stirrer (Corning, France) and homogenized with a sonicator (Sonics Materials Inc., USA). The duration of sonication was adapted to LA concentrations to obtain a similar particle size and, thus minimize textural influences (8). In all cases, sonication was conducted by lapses of 30 s separated by a 1 min pause. Sonication was conducted in a hermetic chamber saturated with nitrogen and beakers were cooled by using an ice bath to limit the formation of oxidized compounds during emulsion preparation.

Samples were presented as 5 ml portions in opaque cups and were tested at room temperature. Subjects were instructed to hold the 5 ml solution in their mouth for 7 s, spit the solution out, and wait for 20 s before tasting the next sample. The interval between 2 sets was 60–120 s, during which participants were asked to rinse their mouths with water. Testing was conducted under red lighting and with participants wearing a nose clip to limit visual and olfactory inputs, respectively. To reduce the potential nutritional interferences on the taste perception due to the last meal before the 3-AFC sessions, all subjects were asked to eat for the dinner, the following standardized meal: raw tomato (100 g) + 1 teaspoon of sunflower oil (5 g), bread (20 g of French baguette), chicken breast (120 g), potatoes cooked in water (250 g), green beans (100 g), butter (5 g), 1 yoghurt sweetened with cane sugar, 1 standard apple compote. All patients indicated that they complied with this protocol in its entirety.

### Statistical analysis

Microbiome analysis: Significant variations in alpha diversity were assessed using the Kruskal–Wallis test or the Wilcoxon rank–sum test. Multidimensional scaling analyses were performed on beta diversity distance matrices and differences between groups were assessed using PERMANOVA and PERMDISP analyses (2,000 permutations). LEfSe analyses were used to determine significant differences in taxa relative abundance between groups (27) using default parameters (alpha parameter significance threshold set to 0.05 and the logarithmic LDA score cutoff set to 2.0).

Other analyses: After a normality test (D’agostino and Pearson), data were analyzed with either an unpaired Mann–Whitney test or a paired Wilcoxon test for paired analysis. Unpaired Mann–Whitney, Wilcoxon tests, and Linear correlation tests were performed using GraphPad Prism (GraphPad Software, USA). Data from 4-days food recall and TOC questionnaires were compared using Student’s test (for means) and Chi-square or Fischer’s exact test (for percentages).

## Results

### Post-VSG changes in body composition and modifications of tongue microbiota surrounding the circumvallate papillae

Mean of BMI and age of the study population were 43.1 ± 0.7 kg/m^2^ and 38.4 ± 1.3 years, respectively. The time-course of the study including the main anthropometric parameters changes are shown in Figure 1A. Six months after VSG, patients displayed a drastic reduction of body weight, BMI, and fat mass associated with a 2-fold decrease of plasma CRP levels (Figure 1B). Analysis of taxonomic diversity at the genus level has pointed out a reduction of CVP microbial richness after VSG (Shannon’s index, Figure 1C) with a composition clearly distinct from that observed before surgery (Bray-Curtis beta-diversity, Figure 1D) as shown by the PERMANOVA test (*p*-value = 0.0005). To identify the differential taxa abundance, a LEfSe analysis with the 16S rRNA gene sequence data was performed (Figure 1E).

**FIGURE 2:**
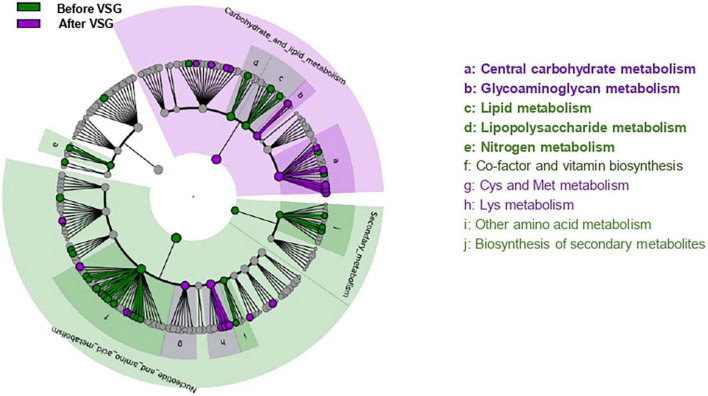
Predicted metagenomics LEfSe changes induced by VSG (before vs. after surgery). Cladogram showing the KEGG metabolic pathway hierarchy represented by rings with the consolidated pathway modules at the outmost ring. KEGG modules are shaded in color according to the group in which it is most abundant. Histogram showing the relative predicted counts for each differential feature with corresponding letters in the cladogram. Numbers at the center of the cladogram correspond to major pathway groupings: 1- Carbohydrate and lipid metabolism; 2- Energy metabolism; 3- Nucleotide and amino acid metabolism; 4- Secondary metabolism. VSG, vertical sleeve gastrectomy.

At the genus level, an enrichment of CVP microbiome in *Prevotella-6, Leptotrichia, Actinomyces*, and *Campylobacter* was found after VSG whereas abundance of *Porphyromonas, Fusobacterium, Neisseria, Prevotella-2, Parvimonas*, and *Pseudomonas* was reduced. Several differential pathways and structural features in the predicted metagenomes were identified from the PICRUSt analysis. LEfSe cladograms of KEGG pathways and structural components derived from pairwise group analysis of pre- and post-VSG patients are shown in Figure 2.

After VSG, the predicted metagenomic profile of microbiota surrounding CVP was notably characterized by a rise of carbohydrate and glycoaminoglycan metabolism along with a decrease in lipid, lipopolysaccharides (LPS) and nitrogen metabolism (Figure 2).

### Post-VSG improvement of LA perception threshold is associated with a specific peripapillae microbiome composition

**FIGURE 3:**
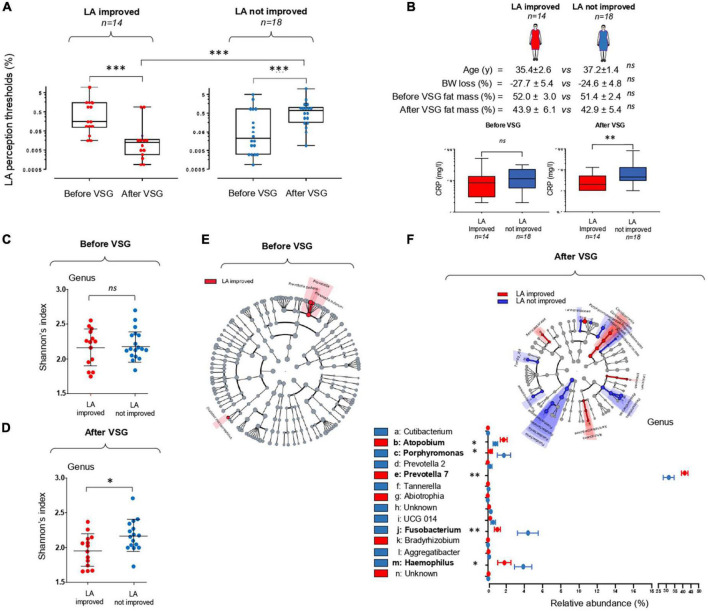
Vertical sleeve gastrectomy (VSG)-associated changes in the orosensory perception of a lipid model (linoleic acid, LA) and in the composition of tongue coating microbiota surrounding the circumvallate gustatory papillae. **(A)** LA perception thresholds before and 6 months after VSG (Mann–Whitney test). **(B)** Comparison of body composition and plasma C-reactive protein (CRP) levels in LA improved and LA not improved patients (Mann–Whitney test). **(C,D)** Comparison of peri-papillae microbiota diversity (Shannon index at the genus level) before and after VSG in LA improved and LA not improved patients. **(E,F)** Taxonomic differential LEfSe analysis before and after VSG on peri-papillae microbiota in LA improved and LA not improved patients. Cladogram of taxonomic assignments from 16S rRNA gene sequence data. Taxa are shaded according to the group in which it is most abundant. Histogram showing the percent relative abundance with corresponding letters in the cladogram. Means ± SEM. *p < 0.05; ^**^p < 0.01; ^***^p < 0.001.

The comparison of LA perception thresholds before and after VSG has allowed distinction of two subgroups: patients displaying a post-operative improvement of the fat taste sensitivity (*i.e.*, a lower LA threshold) and unimproved patients (Figure 3A). This difference of oral lipid sensing was found to be independent of the body weight and fat mass loss after surgery, but was associated in the LA-improved group with a significant reduction of plasma CRP levels, used as a marker of the systemic inflammatory status (28; Figure 3B).

**FIGURE 4:**
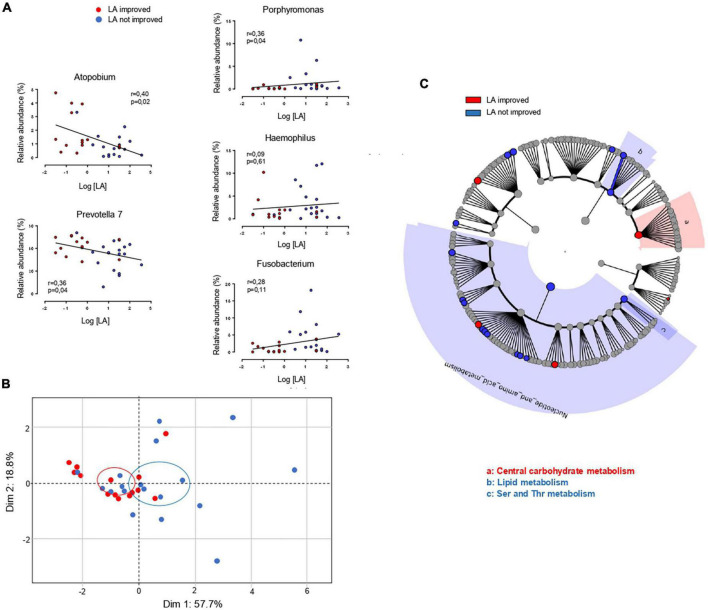
Comparison of oral microbiota in linoleic acid (LA)-improved (*n* = 14) and LA-unimproved (*n* = 18) patients 6 months after the vertical sleeve gastrectomy (VSG). **(A)** Linear regression analyses between the frequency of the mentioned bacterial genus and orosensory perception thresholds of LA. **(B)** Principal component analysis of relationships between microbial profiles and LA perception thresholds 6 month after VSG LA. **(C)** Predicted metagenomics LEfSe changes induced 6 months after a VSG in LA improved and not improved patients. Cladogram showing the KEGG metabolic pathway hierarchy represented by rings with the consolidated pathway modules at the outmost ring. KEGG modules are shaded in color according to the group in which it is most abundant.

To explore the relationship between peripapillae microbiota composition and orosensory perception of LA, bacterial diversity and taxonomy were compared in improved and unimproved patients. In contrast to what was found before surgery (Figure 3C), a statistically significant difference in alpha diversity discriminated LA improved from unimproved patients 6 months after VSG, bacterial richness at the genus level being reduced in LA improved subjects (Figure 3D). Similarly, whereas the taxonomic LEfSe analysis failed to reveal any difference between patient subgroups before VSG (Figure 3E), LA improved patients were characterized by a greater abundance of *Prevotella 7* and *Atopobium* associated with a reduction of *Porphyromonas, Fusobacterium*, and *Haemophilus* bacteria (Figure 3F). To gain insight into relationships between these taxonomic differences and LA perception thresholds, linear regression analyses were conducted after surgery. As shown in Figure 4A, significant negative correlations were found between LA perception thresholds and *Atopobium* (*r* = 0.40, *p* = 0.02) and *Prevotella 7* (*r* = 0.36, *p* = 0.04), a high bacteria level being associated with a low threshold and, thus, a high lipid sensitivity. An opposite scenario was found in patients with *Porphyromonas* (*r* = 0.36, *p* = 0.04–Figure 4A). The multivariate analysis (principal component analysis–PCA) revealed the existence of a strong relationship between the studied variables, the values on the *x*- and *y*-axes, describing the component score of the dimensions 1 and 2, accounting for 57.7 and 18.8% of inertia respectively, (total 76.5%, Figure 4B). Confidence ellipse analysis suggested that the oral microbiota composition in the direct vicinity of CVP was sufficient to distinguish LA-improved from unimproved patients. In line with this assumption, the metagenomic analysis highlighted that the peri-papillae microbiota from LA-improved patients was predominantly characterized by a high carbohydrate metabolic activity while it was mainly oriented towards lipids in unimproved patients (Figure 4C).

Six months after surgery, the daily energy intake was reduced in a same proportion in the two sub-groups (−55.1 and 52.0% in LA-improved and LA-unimproved patients, respectively), the daily carbohydrate, lipid and protein intakes being overall similar (Table 1). Whether a comparable change of sweet, salty, and bitter taste intensity and olfactory performance was reported by the two groups of patients, LA-improved patients displayed a more marked upward trend for the fat taste intensity change than unimproved ones (*p* = 0.04–Table 2).

**TABLE 1:** Reported food intake (4-days food recall) before (*pre-op*) and 6 months (*post-op*) after surgery.

Variables	LA-improved (*n*=14)		LA-unimproved (*n*=18)		LA-I vs. LA-U (post-op)
**Food intake *(g/d)***					
*Pre-op*	1145.6± 156	***	1193.6± 271	***	*ns*
*Post-op*	570.1± 184		567.5± 219		
**Energy intake *(Kcal/d)***					
*Pre-op*	1589.1± 237	***	1545.4± 308	***	*ns*
*Post-op*	875.5± 314		804.3± 191		
**Carbohydrates (% energy intake)**					
*Pre-op*	45.4± 5.3	*ns*	46.3± 6.4	*ns*	*ns*
*Post-op*	44.2± 9.7		43.6± 7.3		
**Lipids (% energy intake)**					
*Pre-op*	34.8± 4.7	*ns*	33.3± 5.6	*ns*	*ns*
*Post-op*	36.6± 9.1		37.9± 6.1		
**Proteins (% energy intake)**					
*Pre-op*	19.8± 2.2	*ns*	20.3± 4.0	*ns*	*ns*
*Post-op*	19.2± 3.1		19.7± 4.9		

Means ± SD; ***p<0.001; ns, not significant. *Pre-op* vs. *post-op*, paired student. I vs. NI, student. Op, operated.

**TABLE 2:** Reported gustatory and olfactory changes [taste and olfaction changes (TOC) questionnaire] 6 months after surgery.

VSG	LA-improved (*n*=14)	LA-unimproved (*n*=18)	*p*
**Taste changes**			
Increased global taste (% of patients)	64.3	50	ns
Intensity of change (1–10)	6.6 ± 1.9	6.2 ± 2.2	ns
Increased sweet taste (% of patients)	71.4	83.3	ns
Intensity of change (1–10)	6.1 ± 2.8	4.1 ± 3.1	ns
Increased fat taste (% of patients)	71.4	66.7	ns
Intensity of change (1–10)	7.7 ± 1.8	5.5 ± 2.9	0.04
Increased salt taste (% of patients)	64.3	38.9	ns
Intensity of change (1–10)	6.3 ± 1.7	4.6 ± 2.9	ns
Increased bitter taste (% of patients)	7.1	16.7	ns
Intensity of change (1–10)	6.0 ± 0.0	6.0 ± 2.0	ns
Olfactory change			
Increased olfaction (% of patients)	35.7	44.4	ns
Intensity of change (1–10)	6.4 ± 1.7	5.3 ± 1.8	ns

Means ± SD; ns, not significant; *p* = X^2^ test of Fisher exact test.

## Discussion

A body of evidence supports the existence of a sixth taste modality devoted to the perception of dietary lipids (26, 29–31). Interestingly, studies report that the taste of fat can be compromised in obesity (7, 31). Although this disturbance may be associated with a preferential consumption of fat-rich foods (8), underlying mechanisms leading to this obesogenic behavior remain poorly understood. As found at the gut level, an oral microbiota dysbiosis is usually observed in subjects with obesity (32). Moreover, an association between the bacterial composition surrounding gustatory papillae and fat taste sensitivity was recently reported in some obese subjects irrespectively from BMI changes (15, 33, 34). The present study strengthens this finding and provides new insights on relationships between obesity surgery and orosensory perception of lipids.

Six months after VSG, the taxonomic diversity at the genus level was found to be reduced in direct vicinity of CVP in comparison to the pre-operative period. This surgery-associated change counteracted the greater alpha diversity currently found in oral microbiota from subjects with obesity (35, 36). Analysis of beta-diversity before and after VSG also revealed a compositional dissimilarity of peri-papillae microbiota communities. Consistent with this data, taxonomic analysis has identified seven bacterial genera, of which the relative abundance was statistically different before and after VSG. By changing the gut microbiota composition, it was previously shown that VSG contributes to the improvement of host metabolism impaired during obesity (37–40). We show herein that VSG is also associated with a remodeling of the tongue microbiome in the direct vicinity of CVP, an event that might affect the host gustatory functions.

Consistent with this assumption, the present study also shows that changes in the orosensory perception of lipids coincide with post-VSG changes in the CVP microbiome. Indeed, CVP microbiota composition was found to be dissimilar between patients with and without improvement of their LA perception thresholds after VSG. Five bacterial genera of which the relative abundance was statistically different were identified. Among these, the level of *Prevotella* genus, considered as a marker of the fiber-rich foods intake (41, 42), was higher in improved patients. Bacteria from *Fusobacterium and Porphyromonas* genera also segregate these two sub-groups, their abundance being higher in LA-unimproved patients suggesting a pro-inflammatory signature. Indeed, cytokines produced by these bacteria are known to promote a local inflammation (43). A pro-inflammatory environment in the direct vicinity of CVP might have a deleterious effect on the fat taste perception by reducing taste bud density and turnover (44), an event known to specifically reduce the fatty taste sensitivity in humans (45). The fact that LA-unimproved patients reported a lower perceived fat intensity than improved patients (Table 2) is consistent with this hypothesis. According to a previous observation done in obese volunteers from another cohort (15, 34), we have found that *Porphyromonas* abundance and LA perception threshold tends to be positively associated after VSG (*i.e.*, high bacteria level = high LA threshold and thus lower LA perception sensitivity). Collectively, these data strongly suggest that VSG might affect the oral perception of lipids by modifying lingual microbiota in some patients.

Although correlation is not causation, it was tempting to speculate that this new microbial balance in direct vicinity of gustatory papillae might participate to the healthier food choice reported by some patients having undergone a VSG (46). Analysis of 4-days food recall questionnaires invalidate this hypothesis. Indeed, similar daily caloric intake and energetic nutrient distribution being found in two sub-groups of patients. Such a disconnection between subjective food sensations and measured oral fat perception has already been found in human and seems to be dependent from methods of investigation used [for a review, (47)].

It is noteworthy that no systematic improvement of the orosensory lipid sensitivity was found after VSG irrespectively of body weight and fat mass loss (Figure 3B). This interindividual variability was already reported in obese subjects (15, 34). Interestingly, a pro-inflammatory bacterial signature in direct vicinity of CVP characterized the obese non-lipid tasters (15). The present study confirms this previous observation and strengthens the hypothesis of an oral microbiota contribution to inter-individual variability of oral lipid detection in human with obesity. However, this phenomenon is likely multiparametric. In previous studies conducted with the same cohort, the plasma Kynurenine levels and the LA perception threshold were found to be positively associated only in the LA- improved group suggesting that Tryptophan/Kynurenine metabolism is one of regulatory factors in the cross-talk between nutritional obesity, bariatric surgery, and fat taste (48). By contrast, despite digestive tract modifications secondary to surgery, any correlation was found between the fat taste sensitivity and the VSG-mediated modifications of main appetite-regulating hormones from the digestive tract, *i.e.*, ghrelin, insulin, GLP-1, and PYY (17).

The main weakness of this study is the low number of patients in each sub-groups (improved, *n* = 14 *vs.* unimproved, *n* = 18). Nevertheless, investigations being performed only in selected women who were their own controls (before *vs.* after VSG) has likely reduced the possible biases due to gender, interindividual variability et taste alterations. Despite this limitation, LA-improved and not improved patients were clearly distinguished after VSG contrary to what is observed before surgery. Another limitation is the use of 4-days food recall to evaluate the eating habits of patients after surgery. Although the self-reporting methods easy to implement are commonly used, they may generate biased responses (47). Studies using a direct method to accurately measure the changes in food selection (e.g., *ad libitum* buffet paradigm (49) will be require to confirm the present data.

In brief, this study shows that VSG is associated with changes in the microbiota composition surrounding the gustatory papillae, this event being coinciding with an improvement of the orosensory perception of lipids in some patients, irrespectively from their BMI. This original observation raises at least two basic questions for future investigations: why this VSG-induced fat taste improvement is not systematically reproducible in human? Does it constitute a long-term health advantage after obesity surgery? Corroboration of present data with larger selection of obese subjects might lead to the validation of easily assayed predictive bacterial biomarkers useful to identify the patients with improved orosensory lipid perception from unimproved patients in order to provide them personalized nutritional advices ensuring the long-term success of the VSG.

## Data availability statement

The data presented in the study are deposited in the ENA repository, accession number PRJEB58719.

## Ethics statement

The studies involving human participants were reviewed and approved by Comité de Protection des Personnes, CPP n° 15-032 and Agence Nationale de la Sécurité des Médicaments, ANSM n° 150811B-21) and registered at Clinical Trials (NCT#02497274). The patients/participants provided their written informed consent to participate in this study.

## Author contributions

PB, SL, and XC: conceptualization. LR, AB, FS, and JC: methodology. PB: formal analysis, writing – original draft preparation, supervision, and funding acquisition. PB, AB, SL, XC, FS, JC, and VB-B: writing – review and editing. AB: project administration. All authors contributed to the article and approved the submitted version.

## Footnotes

1
http://picrust.github.io/picrust/tutorials/genome_prediction.html#genome-prediction-tutorial

2
http://www.genome.jp/kegg/module.html
